# Physical properties, antioxidant capacity, and starch digestibility of cookies enriched with steam-exploded wheat bran

**DOI:** 10.3389/fnut.2022.1068785

**Published:** 2022-12-07

**Authors:** Feng Kong, Yue Li, Di Xue, Yishuai Ding, Xiaofan Sun, Xingfeng Guo, Wenhao Wang

**Affiliations:** ^1^Agricultural Science and Engineering School, Liaocheng University, Liaocheng, China; ^2^Gambol Pet Group Co., Ltd., Liaocheng, China

**Keywords:** steam explosion, solvent retention capacity, antioxidant activity, spread ratio, starch digestibility

## Abstract

Wheat bran-based food is rich in bioactive compounds, and steam explosion enhances the nutritional properties of wheat bran. This study examined the potential utilization of steam-exploded wheat bran (SWB) in cookie formulation. The influence of steam explosion on the chemical compounds in wheat bran and the effects of SWB on the physical properties, antioxidant capacity, and starch digestibility of cookies were investigated. The results showed that steam explosion facilitated the release of reducing sugar, flavonoids, phenolic substances, and amino acid nitrogen in wheat bran, thereby improving its nutritional properties. The reduction of sugar, total flavonoids, total phenolics, and amino acid nitrogen contents of wheat bran after steam explosion increased by 34.22, 183.02, 284.09, and 93.39%, respectively, compared with those of native wheat bran. Substitution of SWB for wheat flour mainly induced higher water, sodium carbonate, and sucrose solvent retention capacities, which were positively related to the spread ratio and hardness of cookies. The cookies with more SWB substitution (30–50%) expressed a higher spread ratio and harder texture than the others. The substitution of SWB caused changes in the antioxidant properties of cookies, which were related to the phenolic content. The cookies with SWB showed a higher DPPH radical scavenging activity (16.30–30.93%) than that of the control (14.74%). SWB might form a matrix barrier to hinder starch digestion, thus reducing the digestibility of cookies. The cookies enriched with 30–50% of the SWB exhibited greater physical properties and antioxidant capacity but lower starch digestibility than those of other cookies. The results will contribute to expanding the application range and improving the quality of bran-rich flour products.

## Introduction

Wheat bran is a major by-product of wheat flour production and is popular in the food industry due to its accessibility and inexpensiveness (1–3). Wheat bran has many health-promoting components, such as dietary fiber and phenolics. Bran fraction has been shown to modulate hunger and satiety moods, influence the glycemic status, and have a prebiotic activity or act as a free radical scavenger (3). Considering the large quantity and developmental nutrition of wheat bran, there have been great efforts to utilize wheat bran in value-added food products. Wheat bran is increasingly being added to flour products due to its multiple physiological effects (4). Despite the health factors promoting wheat bran intake, there are technological and nutritional challenges to using it. The lower bioavailability and shorter shelf life restricted its wide development and utilization. Therefore, improving the storage properties and bioavailability is an efficient strategy to promote wheat bran consumption.

Steam explosion is a technology that modifies materials by hydrothermal and mechanical actions, which has aroused an increased interest in grain processing (5–7). A steam explosion has the potential to disrupt the cell wall structure, thus promoting the release of intracellular components (8–11). The soluble dietary fiber and phenolic contents of wheat bran were significantly increased by steam explosion, which was conducive to improving the antioxidant and antiproliferative properties (8, 11, 12). Additionally, steam explosions effectively inactivated the lipase and peroxidase of wheat bran, improving its storage properties (9). Furthermore, steam explosions effectively reduced the phytic acid content of wheat bran (10). Wheat bran pretreated by the steam explosion was a critical step for the flavor improvement of non-enzymatic browning products (13). Therefore, steam-exploded wheat bran could serve as a suitable ingredient in flour products with good nutritional properties.

As a traditional food, cookies are popular around the world with increasing daily demands; however, manufacturing cookies with refined wheat flour generates a lack of bioactive components with health benefits (14). This study examined the potential utilization of steam-exploded wheat bran as a partially substituted portion of wheat flour in the formulation of bran-based cookies. The influence of steam-exploded wheat bran on solvent retention capacity, colorful profiles, physical properties, antioxidant activity, and starch digestibility of cookies was evaluated.

## Materials and methods

### Materials

Wheat bran was purchased from Chenxi Organic Feed Business Department (Anhui, China). Wheat flour was purchased from Xinxiang Liangrun Whole Grain Food Co. Ltd. (Henan, China), and α-Amylase (35 U/mg) and amyloglucosidase (1 × 10^5^ U/mL) were purchased from Shanghai Macklin Biochemical Co., Ltd. (Shanghai, China).

### Preparation of steam-exploded wheat bran

The wheat bran was treated by a steam explosion at 0.8 MPa for 5 min, as described in the previous reports (8, 15). Moreover, steam-exploded wheat bran was collected and dried at 60°C for 12 h before being ground (100 g) for 2 h to obtain steam-exploded wheat bran powder.

### Chemical compounds of wheat bran

Protein content in native and steam-exploded wheat bran was measured using the AACC method 46–08. Reducing sugar content and amino acid nitrogen content in native and steam-exploded wheat bran was analyzed using the method (13) with modifications. The wheat bran samples (2 g) were dissolved in distilled water (40 mL), equilibrated in a water bath at 25°C for 1 h with constant oscillation, and then centrifuged at 3,000 rpm for 5 min. The collected supernatant (2 mL) was mixed with distilled water (10 mL), and then methyl red, sulfuric acid, and formaldehyde were added. The reduction of the sugar content of wheat bran was measured by 3,5-dinitrosalicylic acid at 540 nm. The Maillard reaction products in the middle and final stages of the non-enzymatic browning reaction were determined at 294 and 420 nm (13).

### Solvent retention capacity of flour blends

The AACC International Approved Method 56-11.02 was used to study the ability of composite flours to hold on to solvents when mixed with different amounts of steam-exploded wheat bran.

### Preparation of cookies

The ingredients of the cookies included flour blends [100 g; steam-exploded wheat bran (0, 10, 20, 30, 40, and 50 g/100 g)], starch (10 g), baking soda (0.5 g), vegetable oil (15 g), white granulated sugar (25 g), table salt (0.5 g), milk (10 g), and water (16 g). Flour blends, starch, and baking soda were mixed uniformly before being mixed with vegetable oil. Table salt and white granulated sugar were dissolved in water and milk, added to the mixture, and mixed to obtain a homogeneous dough. The dough was kneaded, sheeted, and cut into circular shapes with a diameter of 40 mm. The sample was baked at 180°C for 15 min and then at 200°C in an oven for 5 min.

### Color measurement of cookies

The colorful profiles of wheat bran and cookies were tested with a CR-10 chromameter (Minolta, Japan) (16); the chroma value (17), total color difference (ΔE) value (17), and browning index (18) were calculated using L^*^, a^*^, and b^*^ values.

### Physical property analysis of cookies

The spread ratio of cookies was calculated by dividing the diameter by the thickness (19). The hardness of the cookies was determined by a texture analyzer (CT3, Brookfield Engineering Laboratories, Inc., Middleboro, MA) using a TA39 probe. Pre-test speed: 2 mm/s, test speed: 0.5 mm/s, distance: 3 mm, trigger force: 5 g.

### Antioxidant property analysis of cookies

The total phenolic contents of the extraction solution from wheat bran and cookies were determined using the method (20). The total flavonoid content of the extraction solution from wheat bran and cookies was determined using the method (21). The DPPH radical scavenging activity of the extraction solution from wheat bran and cookies was analyzed using a previous method (8).

### *In vitro* starch digestibility of cookies

The previous report determined the *in vitro* starch digestibility of cookies (22). The digestion reaction was terminated at 30 and 60 min, and the glucose content of the digestive juice was measured.

### Statistical analysis

All of the tests were performed at least in triplicate. Experimental data were processed by one-way analysis of variance using IBM SPSS Statistics 20 (IBM, NY, USA) with Duncan's multiple range test (*p* < 0.05). Pearson's correlation and principal component analysis were processed using Origin 2021 software (OriginLab Corporation).

## Results and discussion

### Chemical indices of native and steam-exploded wheat bran

The protein, amino acid nitrogen, reduction in sugar, total phenolics, and total flavonoid contents of wheat bran are listed in Table 1. The results showed an increase in amino acid nitrogen, reducing sugar, total phenolics, and flavonoid contents in steam-exploded wheat bran but no apparent change in protein content. The thermochemical reactions and mechanical actions during the steam explosion process promoted the conversion of insoluble dietary fiber to soluble dietary fiber. The rupture of the cell wall promoted the exposure of intracellular components (11, 23). The cell wall disruption effect of the steam explosion was conducive to enhancing the amino acid nitrogen (93.39%), reducing sugar (34.22%), phenolics (284.09%), and flavonoids (183.02%) contents compared to the native wheat bran. Furthermore, steam explosion enhanced the bile salt adsorption capacity, cholesterol adsorption capacity, antioxidant capacity, and antiproliferative property of wheat bran (8, 9, 12, 13). These characteristics of steam-exploded wheat bran might enhance the nutritional value of the final flour products.

**Table 1:** Chemical compounds of native and steam-exploded wheat bran.

**Sample**	**Protein (%)**	**AAN (mg/g)**	**RS (mg/g)**	**TPC (mg/g)**	**TFC (mg/g)**
NWB	18.01 ± 1.25	4.39 ± 0.32	2.63 ± 0.16	0.44 ± 0.08	2.65 ± 0.05
SWB	17.55 ± 1.26	8.49 ± 0.70^#^	3.53 ± 0.14^#^	1.69 ± 0.08^#^	7.50 ± 0.07^#^

NWB, native wheat bran; SWB, steam-exploded wheat bran; AAN, amino acid nitrogen; RS, reducing sugar; TPC, total phenolic content; TFC, total flavonoid content (^#^p < 0.05).

The changes in the color of wheat bran during the steam explosion are shown in Table 2. The whiteness (L^*^ value) of wheat bran decreased significantly by a steam explosion from 37.83 to 30.20, while the browning index increased from 127.07 to 174.07, indicating that wheat color bran became darker. Compared with native wheat bran, the intermediate product and final product of steam-exploded wheat bran at 294 and 420 nm were increased by 11.02 and 119.85%, indicating that steam explosion supported the accumulation of Maillard reaction products (6, 10, and 13). The steam explosion changed the color profiles of wheat bran from faint yellow to brown (11), which might be useful in cookies (24). The steam explosion fortified the nutritional characteristics of wheat bran. For producing healthy bran-based cookies, steam-exploded wheat bran might be used as one of the potential alternatives to wheat flour. The technical properties of steam-exploded wheat bran and its effects on cookie quality were discussed in the following experiments:

**Table 2:** Colorful profiles of native and steam-exploded wheat bran.

**Sample**	L*	a*	b*	**BI**	**A_294nm_**	**A_420nm_**
NWB	37.83 ±0.15	29.37 ± 0.32	20.33 ± 0.12	127.07 ± 0.70	1.18 ± 0.01	1.31 ± 0.02
SWB	30.20 ± 0.62^#^	26.73 ± 0.21^#^	21.87 ± 0.59^#^	174.07 ± 3.10^#^	1.31 ± 0.02^#^	2.88 ± 0.02^#^

NWB, native wheat bran; SWB, steam-exploded wheat bran; A_294nm_ and A_420nm_ indicated the absorbance at 294 and 420 nm; BI, browning index (^#^p < 0.05).

### Solvent retention capacity of composite flours

**Figure 1:**
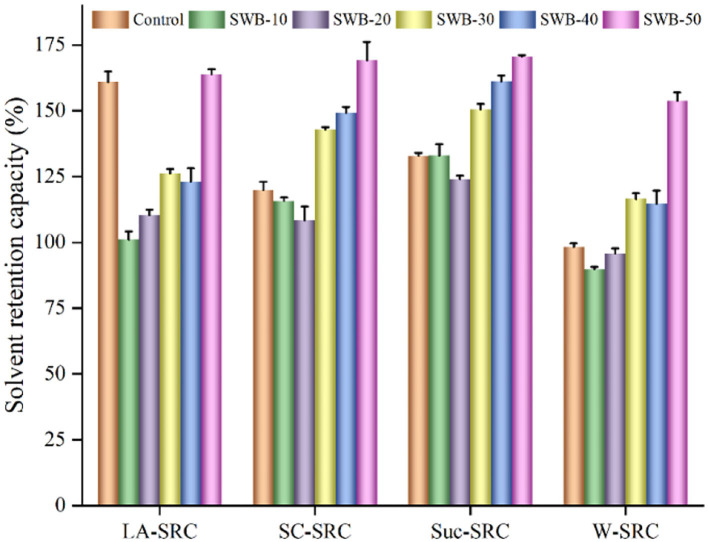
Effect of steam-exploded wheat bran on the solvent retention capacity of flour blends. SWB: steam-exploded wheat bran; SWB-10 to SWB-50 indicated that the amount of steam-exploded wheat bran was 10–50 g/100 g flour blend, respectively. SRC, solvent retention capacity; LA-SRC, SC-SRC, Suc-SRC, and W-SRC, solvent retention capacity of lactic acid, sodium carbonate, sucrose, and water, respectively.

Solvent retention capacity was an efficient indicator to evaluate the quality of wheat flour and the effects of wheat flour on the quality of cookie products (25, 26). The observed differences in solvent retention capacity between control and composite flours incorporated with steam-exploded wheat bran (Figure 1). The solvent retention capacity values of control were as follows: lactic acid solvent retention capacity = 160.93 g/100 g, sodium carbonate solvent retention capacity = 119.86 g/100 g, sucrose solvent retention capacity = 132.87 g/100 g, and water solvent retention capacity = 98.26 g/100 g. The flour blends substituted with 30–50% of the steam-exploded wheat bran significantly increased the water, sodium carbonate, and sucrose solvent retention capacity values. Water solvent retention capacity increased significantly with the addition of steam-exploded wheat bran due to the higher water-holding capacity of wheat bran and dietary fiber, which had more hydrophilic groups. Sucrose solvent retention capacity reflected pentosan/arabinoxylans content, and the sucrose solvent retention capacity values of flour blends increased because of the high arabinoxylans content in the steam-exploded wheat bran. By substitution, the phenolic and dietary fiber concentrations of the flour blends significantly increased with the increasing amounts of steam-exploded wheat bran, which might affect the physical and healthy quality of the final products.

### Color values of steam-exploded wheat bran-substituted cookies

The substitution of steam-exploded wheat bran for wheat flour impacted the colorful profiles of the cookies (shown in Table 3). Adding steam-exploded wheat bran to the formulations of cookies triggered the decrease of L^*^ and b^*^ values, whereas it increased the ^*^ value of the cookies. Color measurements indicated that cookies incorporated with steam-exploded wheat bran yielded a darker color (lower L^*^ values) (27). The ^*^ values of the cookies substituted with steam-exploded wheat bran ranged from 10.93 to 13.06, which was noticeably different from the control cookie. The b^*^ values of the cookies were evidently (*p* < 0.05) decreased by steam-exploded wheat bran compared with that of the control. Higher protein and fiber content might enhance the formation of Maillard reaction products, which was conducive to a decline in the lightness and an increase in the redness of cookies after baking (28). Similar results were found in cookies made with cellulase-treated wheat bran (14). The total color difference (Δ?) of cookies enriched with steam-exploded wheat bran varied from 11.49 to 30.13. The chroma values of cookies indicated their purity or saturation (17); no change was found in chroma between the control and cookies incorporated with 10% steam-exploded wheat bran (*p* > 0.05). The hue angle values of cookies decreased from 1.31 to 1.11 by adding steam-exploded wheat bran. There was an obvious negative correlation (*p* < 0.01) between L^*^ and ΔE (*r* = −1.000), L^*^ and a^*^ (*r* = −0.988), a^*^ and b^*^ (*r* = −0.943), a^*^ and Hue (*r* = −0.989), a^*^ and Chroma (*r* = −0.893), b^*^ and ΔE (*r* = −0.977), while L^*^ and b^*^ (*r* = 0.975), L^*^ and Hue (*r* = 0.998), L^*^ and Chroma (*r* = 0.938), a^*^ and ΔE (*r* = 0.988), b^*^ and Hue (*r* = 0.980), b^*^ and Chroma (*r* = 0.991), showed a remarkably positive relationship (*p* < 0.01).

**Table 3:** Effect of steam-exploded wheat bran on the chrominance of cookies.

**Samples**	L*	a*	b*	**ΔE**	**Hue**	**Chroma**
Control	74.40 ± 1.47^a^	8.63 ± 0.78^d^	31.90 ± 1.14^a^	0.00 ± 0.00^e^	1.31 ± 0.02^a^	33.05 ± 1.29^a^
SWB-10	63.27 ± 2.75^b^	10.93 ± 0.87^c^	30.37 ± 0.80^b^	11.49 ± 3.50^d^	1.23 ± 0.02^b^	32.28 ± 1.05^a^
SWB-20	59.97 ± 0.76^c^	10.93 ± 0.50^c^	28.37 ± 0.76^c^	15.15 ± 1.86^c^	1.20 ± 0.01^c^	30.40 ± 0.89^b^
SWB-30	51.90 ± 0.70^d^	11.90 ± 0.26^bc^	27.33 ± 0.46^dc^	23.20 ± 1.53^b^	1.16 ± 0.01^d^	29.81 ± 0.50^b^
SWB-40	47.83 ± 1.05^e^	12.73 ± 0.12^ab^	27.10 ± 0.36^cd^	27.34 ± 1.17^a^	1.13 ± 0.02^e^	29.94 ± 0.36^b^
SWB-50	45.13 ± 0.85^f^	13.06 ± 0.49^a^	26.40 ± 0.26^d^	30.13 ± 0.92^a^	1.11 ± 0.07^e^	29.46 ± 0.38^b^

SWB, steam-exploded wheat bran; SWB-10 to SWB-50 indicated the amount of steam-exploded wheat bran was 10–50 g/100 g flour blend, respectively. Different letters indicated significant differences at p < 0.05 in the same column.

### Physical properties of cookies incorporated with steam-exploded wheat bran

**Figure 2:**
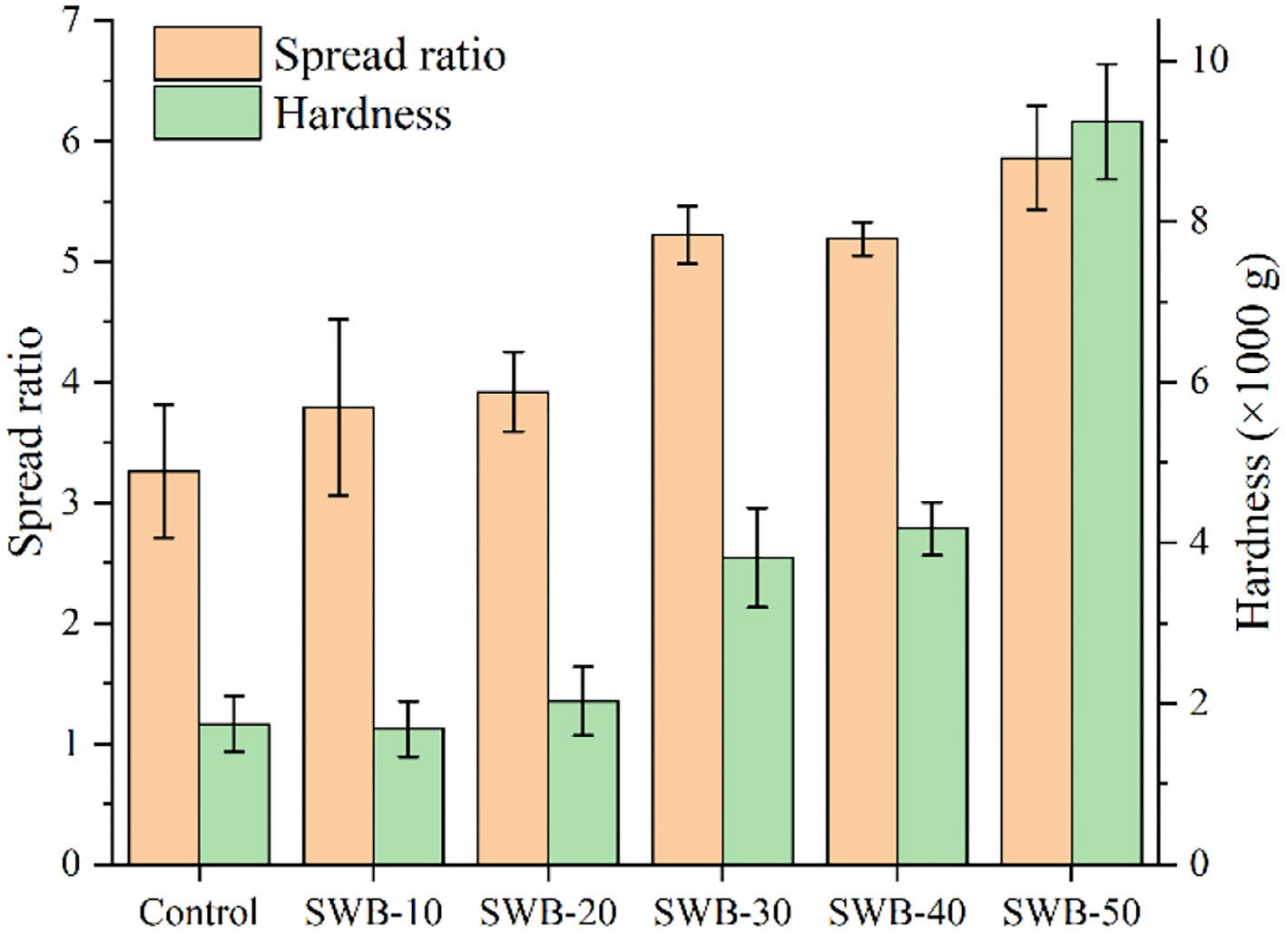
Effect of steam-exploded wheat bran on the physical properties of cookies. SWB, steam-exploded wheat bran; SWB-10 to SWB-50 indicated that the amount of steam-exploded wheat bran was 10–50 g/100 g flour blend, respectively.

The physical properties of cookies, including spread ratio and hardness, as affected by the addition of steam-exploded wheat bran, are shown in Figure 2. The spread ratio and hardness of the cookies varied from 3.26 to 5.89 and 1,684.89 to 9,241.56 g, respectively. High-quality cookie flour was usually associated with a higher spread ratio (29). A higher spread ratio usually indicates better quality parameters of cookies, which might be preferable to consumers (30). The influence of the substituted ratios (0–20%) of steam-exploded wheat bran on the physical properties of cookies was insignificant. The spread ratio of cookies was significantly increased by substituting 30–50% of steam-exploded wheat bran for wheat flour. The high spread ratio of cookies might be responsible for the dilution effect of steam-exploded wheat bran on the gluten (31). Compared to the control cookie, the hardness of the cookies dramatically increased with the addition of steam-exploded wheat bran, from 30 to 50%. Steam-exploded wheat bran brought the harder texture of the cookie, which could be responsible for the elevated content of insoluble dietary fiber and protein and the enhanced water-holding capacity of the composite flours (14). Therefore, steam-exploded wheat bran might be suitable for producing cookies with enhanced textural quality.

### Antioxidant properties of cookies enriched with steam-exploded wheat bran

**Figure 3:**
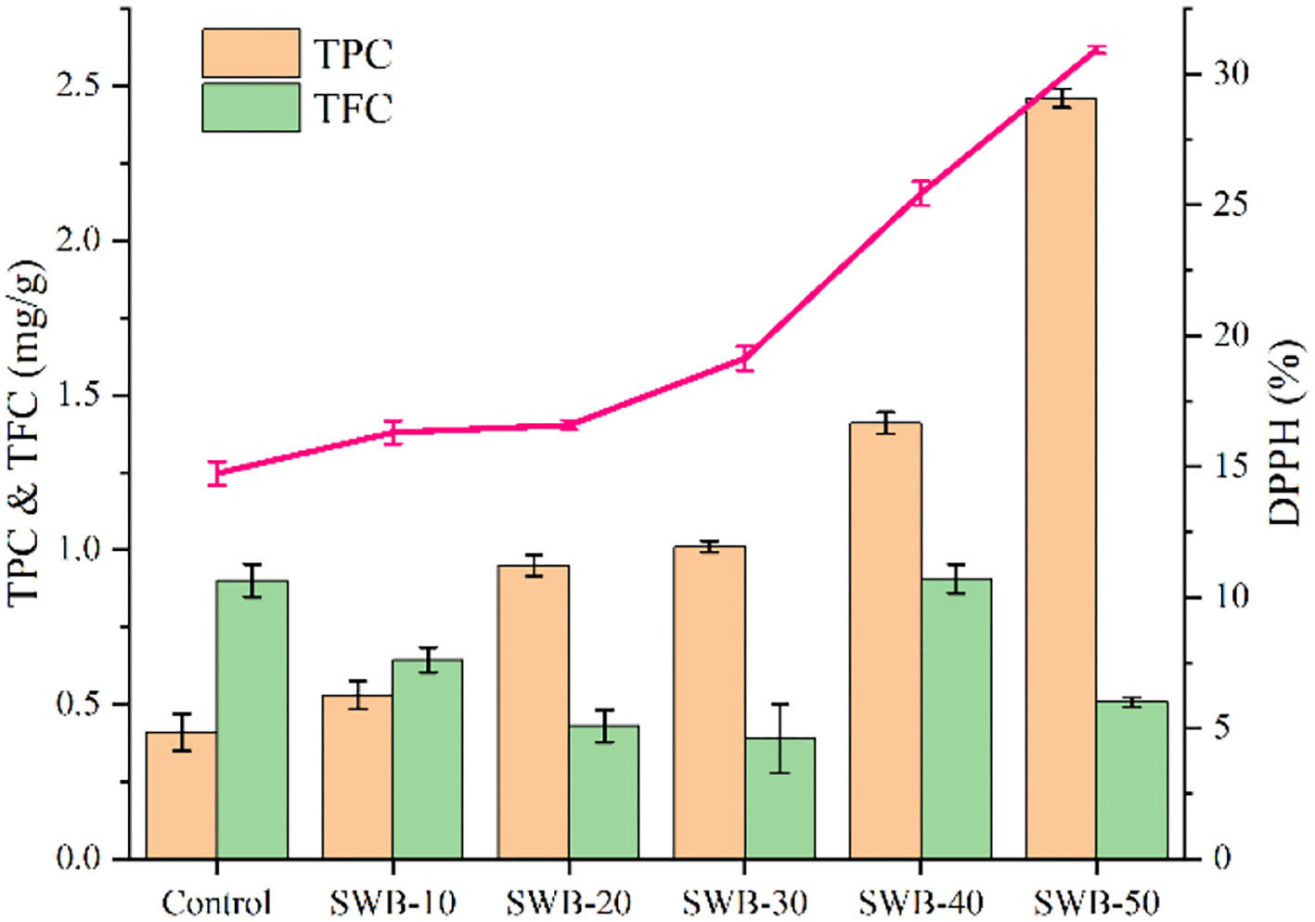
Effect of steam-exploded wheat bran on the antioxidant property of cookies. TPC, total phenolic content; TFC, total flavonoid content; DPPH, DPPH radical scavenging activity; SWB, steam-exploded wheat bran; SWB-10 to SWB-50 indicated the amount of steam-exploded wheat bran was 10–50 g/100 g flour blend, respectively.

The antioxidant properties of cookies incorporated with steam-exploded wheat bran were determined according to the total phenolic content, flavonoid content, and DPPH radical scavenging capacity. As shown in Figure 3, cookies incorporating steam-exploded wheat bran had a higher total phenolic content and DPPH radical scavenging activity. Total phenolic content increased with the increasing amounts of steam-exploded wheat bran in the flour blends, ranging from 0.41 to 2.46 mg/g. There was no correlation between the total flavonoid content of cookies and the amount of steam-exploded wheat bran. The presence of reduced sugars, Maillard reaction products, and proteins may cause changes in flavonoids during baking (32, 33). However, the mechanism of flavonoid content changes induced by steam-exploded wheat bran is unclear. The extract from cookies incorporated with steam-exploded wheat bran showed a higher DPPH radical scavenging activity (16.30–30.93%) than that of the control cookie (14.74%). The total phenolic content of cookie extracts exhibited a significant correlation with the DPPH radical scavenging activity (*p* < 0.05, *r* = 0.965). Adding fiber-rich and phenolic compounds to foods had physiological effects on blood cholesterol levels and positive effects on sensory properties (34). Furthermore, the baking process enhances the DPPH radical scavenging activity of cookies due to the formation of Maillard reaction products, which have antioxidant properties (35).

### Starch digestibility of the cookies substituted with steam-exploded wheat bran

**Figure 4:**
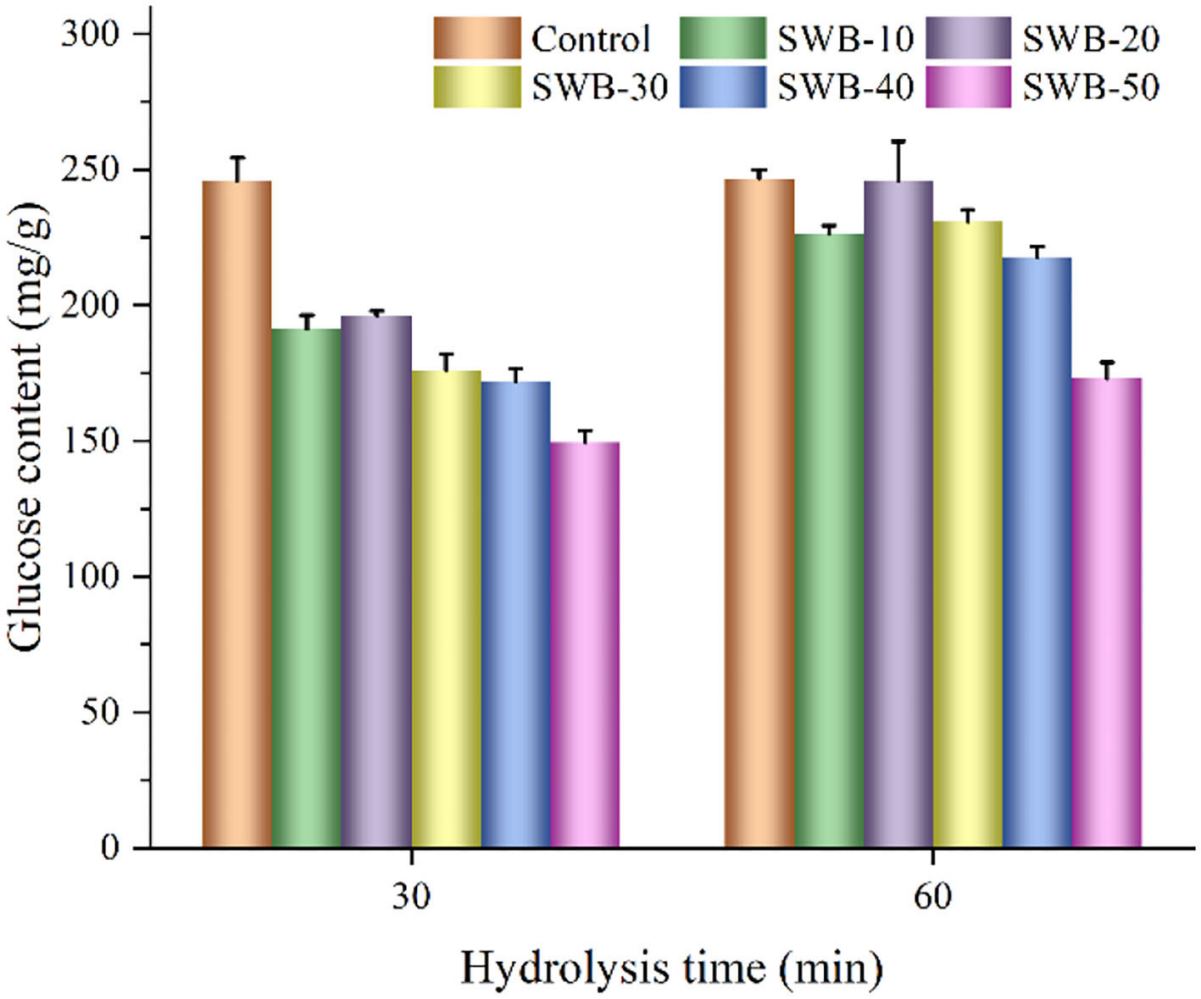
Glucose content in digestive juice after starch digestion of cookies. SWB, steam-exploded wheat bran; SWB-10 to SWB-50 indicated the amount of steam-exploded wheat bran was 10–50 g/100 g flour blend, respectively.

The glucose content of digestive juice after enzymatic digestion is shown in Figure 4. The glucose content was affected by the substituted ratios of steam-exploded wheat bran. The cookies enriched with steam-exploded wheat bran showed lower glucose content (149.44–196.16 mg/g) and the hydrolysis rate of starches in the first 30 min compared to the control cookie (245.89 mg/g). From 30 to 60 min, there was a noticeable drop in the hydrolysis rate for cookies. The ultimate glucose content of the control cookie was 246.79 mg/g, while that of cookies incorporating steam-exploded wheat bran was 173.08–245.93 mg/g. The amount of steam-exploded wheat bran at 50% resulted in the lowest ultimate glucose content (173.08 mg/g) of cookies. These results were possibly due to the dietary fiber and protein in steam-exploded wheat bran, which formed a physical barrier that enclosed the starch granules and caused the starch to be hindered by digestion (31); this finding might be attributed to the cookies having more dietary fiber while having less starch content; the cookie enriched with steam-exploded wheat bran might be used as an ideal functional food product (14).

### Correlation and principal component analysis of various parameters of cookies

**Figure 5:**
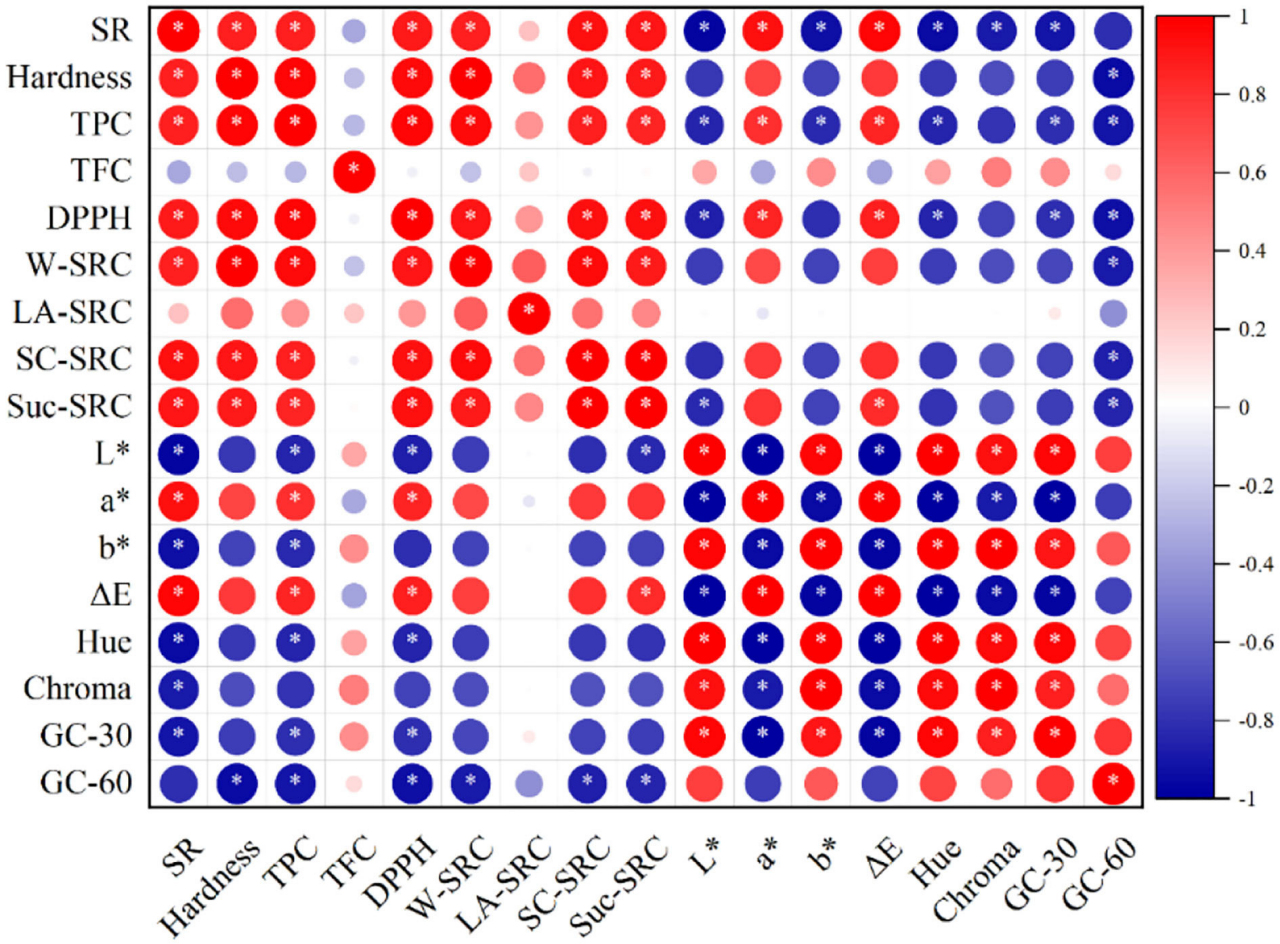
Pearson's correlation among various parameters of cookies. SR, spread ratio; TPC, total phenol content; TFC, total flavonoid content; DPPH, DPPH radical scavenging activity; GC-30, glucose content at 30 min; GC-60, glucose content at 60 min; SRC, solvent retention capacity; LA-SRC, SC-SRC, Suc-SRC, and W-SRC, solvent retention capacity of lactic acid, sodium carbonate, sucrose, and water, respectively. *p<0.05.

Elucidating the correlations between the colorful profiles, antioxidant properties, physicochemical characteristics, and starch digestibility of the cookies shown in Figure 5 might be conducive to developing a formula to control the quality according to the substituted amount changes of steam-exploded wheat bran. The total phenolic content of cookie extracts exhibited significant (*p* < 0.05) positive correlations with the DPPH radical scavenging activity. Total phenolic content showed a strong negative relationship with the L^*^, b^*^, and Hue values, while total phenolic content positively correlated with the ^*^ and ΔE values. The spread ratio was positively related to hardness (*r* = 0.87), water solvent retention capacity (*r* = 0.88), sodium carbonate solvent retention capacity (*r* = 0.92), and sucrose solvent retention capacity (*r* = 0.92). In contrast, it was negatively connected to the color parameters (L^*^, b^*^, hue, and chroma values). Hardness was positively related to the spread ratio, water solvent retention capacity, sodium carbonate solvent retention capacity, and sucrose solvent retention capacity. The glucose content at 60 min was negatively related to the hardness, total phenolics, DPPH radical scavenging activity, water, sodium carbonate, and sucrose solvent retention capacity.

**Figure 6:**
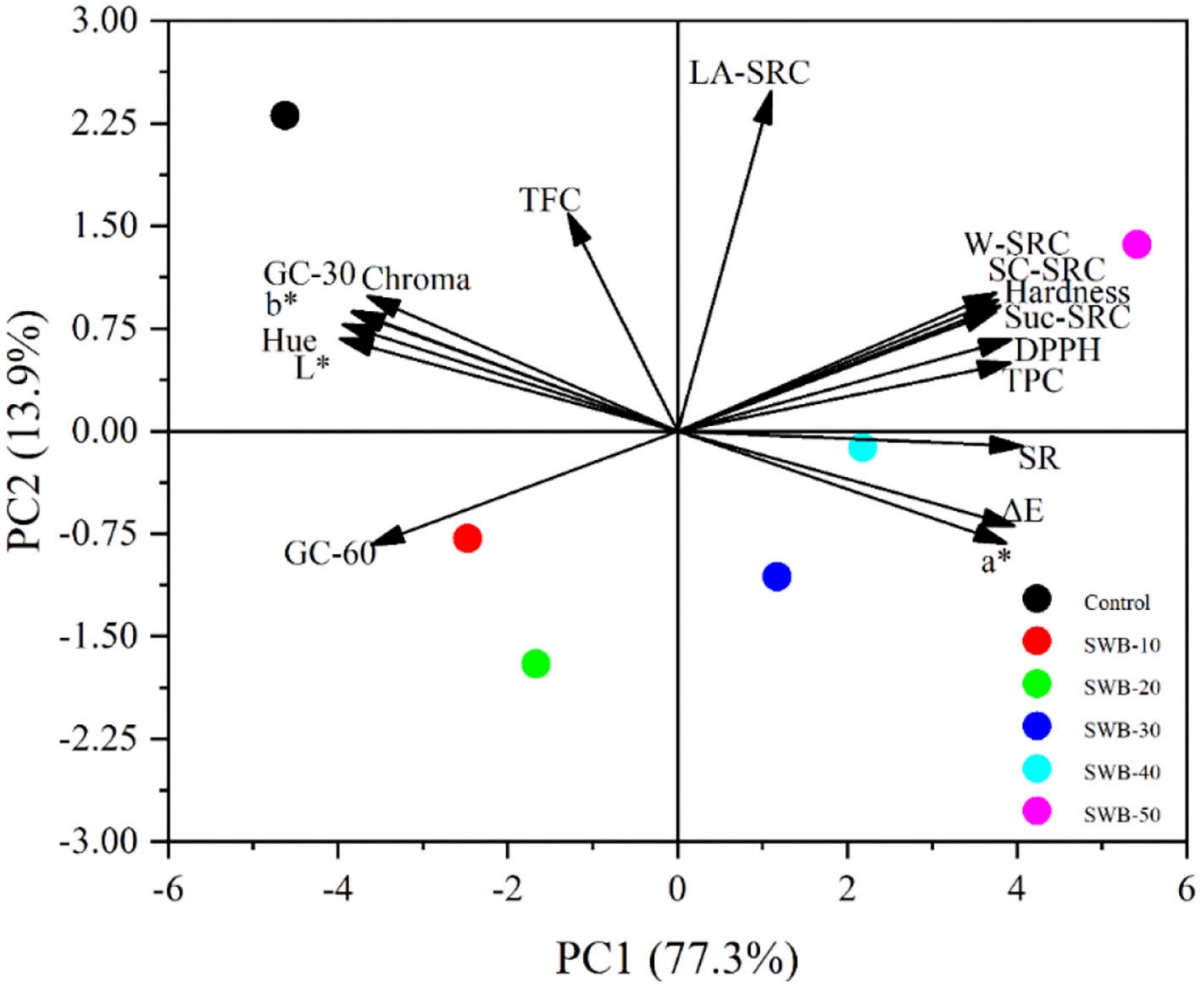
The PCA of the various parameters of cookies. SR, spread ratio; TPC, total phenol content; TFC, total flavonoid content; DPPH, DPPH radical scavenging activity; GC-30, glucose content at 30 min; GC-60, glucose content at 60 min; SWB, steam-exploded wheat bran; SWB-10 to SWB-50 indicated the amount of steam-exploded wheat bran was 10–50 g/100 g flour blend, respectively; SRC, solvent retention capacity; LA-SRC, SC-SRC, Suc-SRC, and W-SRC, solvent retention capacity of lactic acid, sodium carbonate, sucrose, and water, respectively.

A principal component analysis (PCA) was constructed to get a complete picture of the effects of steam-exploded wheat bran on the quality of cookies. The PCA shown in Figure 6 exhibited 77.3 and 13.9% of the variability for the first principal component (PC1) and second principal component (PC2), respectively. The PC1 was characterized by major positive levels of solvent retention capacity, hardness, spread ratio, total phenolic content, and DPPH radical scavenging activity and major negative levels of total flavonoid content, glucose content, and color parameters L^*^, b^*^, hue, and chroma. For the PC2, the glucose content at 60 min, a^*^, and ΔE showed negative values, whereas other properties showed positive values. The remarkable diversity of the functionality among the different substituted ratios of steam-exploded wheat bran was represented by the PCA that was distinguished from all the parameters measured. The control cookie made from wheat flour had a higher glucose content, L^*^, b^*^, hue, and chroma values. Conversely, cookies incorporating 50% steam-exploded wheat bran led to higher water, sodium carbonate, sucrose solvent retention capacity, spread ratio, hardness, total phenolic content, DPPH radical scavenging activity, and a^*^ and ΔE values. However, cookies substituted with steam-exploded wheat bran from 10 to 40% showed intermediate results.

## Conclusion

The effects of wheat bran modification by a steam explosion on chemical characteristics and the desirable quality of the cookies substituted with steam-exploded wheat bran for wheat flour were investigated. Steam explosion modified wheat bran by facilitating the release of flavonoids and phenolic substances, the reduction of sugar and amino acid nitrogen, and thus improving its nutritional properties. Substitution of steam-exploded wheat bran for wheat flour mainly led to higher water, sodium carbonate, and sucrose solvent retention capacities, which were positively related to cookies' spread ratio and hardness. The addition of steam-exploded wheat bran increased the antioxidant property and decreased the starch digestibility of cookies. The study demonstrated that nutritional and healthy cookies could be prepared by incorporating steam-exploded wheat bran. Steam explosion improved the nutritional characteristics and feasible utilization of wheat bran, and steam-exploded wheat bran could serve as a potential ingredient in flour products.

## Data availability statement

The original contributions presented in the study are included in the article/supplementary material, further inquiries can be directed to the corresponding author.

## Author contributions

Conceptualization, writing-original draft preparation, writing-review and editing, supervision, project administration, and funding acquisition: FK. Investigation: FK, YD, DX, and XS. Resources: FK, WW, and XG. All authors have read and agreed to the published version of the manuscript.
